# Establishment of Breast Cancer Organoids: A Systematic Review and Meta‐Analysis

**DOI:** 10.1155/ijbc/6534449

**Published:** 2026-05-10

**Authors:** Vinícius Marques Rocha, Maria Lucia Hirata Katayama, Larissa Oliveira Amorim, Beatriz Domenici de Oliveira, Rossana Veronica Mendoza Lopez, Maria Aparecida Azevedo Koike Folgueira

**Affiliations:** ^1^ Faculdade de Medicina, Universidade de São Paulo (FMUSP), São Paulo, Brazil, usp.br; ^2^ Comprehensive Center for Precision Oncology (C2PO), Translational Oncology Investigation Center (CTO), Cancer Institute of the State of São Paulo (ICESP), Clinics Hospital (HCFMUSP), Faculdade de Medicina, Universidade de São Paulo, São Paulo, Brazil, usp.br; ^3^ Cancer Institute of the State of São Paulo (ICESP), Hospital das Clínicas (HCFMUSP), Faculdade de Medicina, Universidade de São Paulo, São Paulo, Brazil, usp.br

**Keywords:** culture media, breast neoplasia, organoids, systematic review

## Abstract

**Background:**

Organoid culture has emerged as a promising model for studying normal and tumor tissues. Organoids may resemble their tissue of origin, allowing assessment of disease behavior and drug response. In breast cancer (BC), successful organoid establishment may depend on factors like time from tissue collection to culture, dissociation methods, and media composition, which may affect tumor growth and tissue similarity across culture passages. This systematic review and meta‐analysis are aimed at assessing the success rate of BC organoid establishment and their similarity to parental tumors.

**Methods:**

Following PRISMA guidelines, we conducted an electronic search in PubMed, Embase, and Web of Science using terms related to “breast cancer” and “organoids”. Heterogeneity was assessed by I2 (%), with forest plots for all studies and subgroups. A fixed‐effects meta‐analysis estimated the overall establishment rate with 95% confidence intervals.

**Results:**

We analyzed 59 full‐text articles. Five culture media types and 37 added components were reported, most frequent EGF (80%), glutamine, FGF, R‐spondin (66%, each), Y27632, and N‐acetylcysteine (64%, each). Ten studies provided data on initial samples and established organoids, totaling 504 and 349, respectively, with an establishment rate of 71.34% (95% CI: 67.55–75.14), varying from 31.25% to 86.21%. By subtype, establishment rates were as follows: Luminal A (57.47%), Luminal B (72.51%), HER2+ (71.24%), and triple‐negative (78.75). Concordance between receptor expression in tumors and organoids was observed, with kappa values of 0.692 for ER, 0.597 for PR, and 0.711 for HER2. However, positive receptor expression reverted to negative in 19%, 31%, and 28% of organoids for ER, PR, and HER2, respectively.

**Conclusion:**

Breast cancer organoids were successfully established from primary tumor samples, demonstrating good overall concordance in receptor expression with the original tumors. These findings support the feasibility of patient‐derived organoids as translational models; however, continuous phenotypic characterization across passages is recommended.

## 1. Introduction

Effective in vitro models that accurately replicate the behavior of breast cancer are essential instruments for cancer research. They facilitate a deeper comprehension of the molecular mechanisms underlying tumor growth, invasion, and metastasis, thereby aiding in the identification of novel cancer targets. One commonly used in vitro model is the monolayer culture, also known as 2D culture, which is popular due to its simplicity and cost‐effectiveness. However, 2D cultures fail to recapitulate the complex cellular interactions and architectural organization of actual tumors, limiting their ability to accurately reflect in vivo conditions [1].

An alternative, more sophisticated model is three‐dimensional (3D) culture, which better mimics the architecture and complexity of organs and tumors. This model bridges the gap between 2D cultures and in vivo models, offering a more realistic representation of tumor behavior [2]. 3D cultures include spheroids, patient‐derived organoids (PDO), and the “organ‐on‐a‐chip” model. These cell cultures may derive from established cell lines but also from freshly collected human tumors or from patient derived xenograft (PDX) that consists of human tumor implanted in immunosuppressed animals. The last two models can maintain tumor architecture and relative heterogeneity and proportion of cancer and stromal cells, better resembling the primary tumor than 2D cultures [3]. However, there are some disadvantages to this type of model. The cost of establishing these grafts is quite high, and the success rate varies according to the tumor subtype [4].

Spheroids, which can be formed from cell lines or patient‐derived cells, are another type of 3D model. They result from the induced or spontaneous aggregation of cells in suspension cultures, resulting in complex, multicellular structures with diverse morphologies [5, 6]. Organoids, on the other hand, are developed from patient tissues. These tissues are dissociated into small fragments, cultured in a matrix enriched with growth factors that subsequently form 3D structures that closely resemble the original organ in both structure and function. The “organ‐on‐a‐chip” model uses a device that mimics vascularization with highly organized microchambers and pneumatic chambers on both sides. The dissociated cells are carried through an inlet into the top chamber, where there is a matrix‐coated porous membrane [5, 7]. Lastly, PDO can be cultured from adult and embryonic stem cells with high efficiency. These organoids show phenotypic aspects similar to their derived organs and have the ability to self‐organize [2, 8].

The original experiments were led by Sato and colleagues, who developed a protocol to establish 3D murine intestinal organoids embedding them in Matrigel, supplemented with a culture medium containing DMEM/F12, EGF, R‐spondin, Noggin, and Y27632. They observed significant similarities between the established culture and the tissue of origin, including the formation of crypts and lumen. These organoids were maintained for over 8 months. After several passages, microarray tests were conducted to analyze gene expression, showing that the organoids maintained expression profiles very similar to those of the original tissue. Since this 2009 study, similar protocols have been used to generate organoids from other tissues such as the stomach, liver, pancreas, and breast [9].

As organoid culture lacks mesenchymal cells, it requires a scaffold, typically utilizing Matrigel, a basement membrane extract purified from the Engelbreth‐Holm‐Swarm mouse sarcoma, primarily composed of laminin and Collagen IV and considered the gold standard for organotypic culture. However, there are studies that solely utilize collagen 3D as a scaffold [10, 11]. Besides the membrane as support, it is necessary to enrich the medium with several factors, various growth factors or inhibitors, such as EGF, R‐spondin (a Wnt agonist), Noggin (a TGF‐*β* inhibitor), nicotinamide, FGF, A83‐01 (an ALK inhibitor), and SB202190 (a p38 mitogen‐activated protein kinase inhibitor); however, these were not a fixed protocol [12–14]. Some protocols have used neuregulin 1/heregulin, an EGF family protein with a range of functions essential for the development of the central nervous system and heart, which increases the growth rate of normal organoids. However, it does not seem necessary for establishing breast tumor organoids. Nevertheless, Guillen et al. [15] utilize it to establish HER2 immunohistochemical subtype organoids, likely due to its association with the HER2 protein family [15–17].

A pivotal contribution to BC organoid establishment was made in 2018 by Sachs et al., who developed a protocol using niche factors such as epidermal growth factor (EGF), the Wnt‐agonist R‐spondin, the TGF‐*β* inhibitor Noggin, and extracellular matrix surrogates like Matrigel. This protocol led to the creation of a biobank of BC organoids [12]. Although various protocols for BC organoid cultivation have been used with differing levels of success, no single model has emerged as the definitive standard. The primary objective of this systematic review was to evaluate the success rates of breast cancer organoid establishment.

The secondary objectives were to:

• Describe the most commonly used culture medium components for establishing breast cancer organoids.
• Assess the success rates of organoid establishment across different immunohistochemical subtypes (Luminal, HER2‐positive, and triple‐negative).
• Compare establishment success rates between patient‐derived and xenograft‐derived breast cancer organoids.
• Evaluate the similarity between organoids and their corresponding reference samples in terms of morphology, cytology, protein expression (ER, PR, and HER2), and copy number alterations.

## 2. Material and Methods

This systematic review was registered in PROSPERO (International Prospective Register of Systematic Reviews with ID: CRD42021287404).

Reporting of this review was done according to the Preferred Reporting Items for Systematic Reviews and Meta‐Analyses (PRISMA) guidelines [18] (Material S1).

### 2.1. Studies Identification

We performed a systematic electronic search in PubMed, Embase, and Web of Science querying for articles describing the establishment of breast cancer organoids. The first search was conducted in August 2021 and included manuscripts published from database inception to August 26, 2021. Afterwards, we performed a second search on January 10, 2023, including the period August 26, 2021 to January 10, 2023 using the same strategy used in the first search. The search strategy included two elements: “organoids” and “breast cancer”, with a wide range of keywords and MeSH terms combinations, adapted for each database, that are shown in Material S2.

### 2.2. Data Extraction

One of the investigator (V.M.R.) downloaded all reference titles into Mendeley, and eliminated duplicates. These remaining references were exported to Rayyan QCRI (https://www.rayyan.ai/) for blinded revision of titles and abstracts in pairs. The selection of potential studies for inclusion was done by four independent investigators (V.M.R., M.L.H.K., B.D.O., and L.O.A.), in pairs, who screened titles and abstracts.

Afterwards, the investigators, in pairs, independently evaluated the full‐text version of each selected study to determine eligibility. Likewise, the four investigators, in pairs, extracted relevant data from the included articles and added them to a Microsoft Excel spreadsheet. In each step, disagreements were discussed in pairs and, if unresolved, they were discussed with the whole study group.

Data on the following aspects were extracted for each reference: (1) first author′s name; (2) year of publication; (3) number of samples used; (4) if the donor received neoadjuvant treatment; (5) whether the sample was from a primary tumor or metastatic site; (6) type of tumor collection (biopsy, surgery, or PDX); (7) tumor histology; (8) tumor subtype; and (9) immunoexpression of ER, PR, HER2, and Ki67 proteins, in the tumor sample and in the established organoid. Concerning the organoids, the following aspects were extracted: (10) whether the organoid was established from fresh tumor or from PDX; (11) if there was any type of cellular selection before organoid establishment; (12) type of cell dissociation method (mechanical or enzymatic); (13) components of the culture medium; and (14) number of cell passages. If the organoid was established from PDX samples, the following information was also extracted: (15) strain of mice; (16) sample processing before grafting; and (17) implantation site. The following data were obtained from the established organoids: (18) if the organoid maintained the characteristics of the original sample; (19) if the organoids were treated; (20) type of treatment; (21) whether the original sample and the organoid were submitted to genomic sequencing; (22) sequencing information; and lastly, (23) establishment rate.

We retrieved immunoexpression of ER, PR, HER2, and Ki67 and used it to infer BC subtypes, considering luminal tumor any expression of ER and/or PR.

### 2.3. Study Selection

Inclusion criteria for the manuscripts were as follows: (1) establishment of organoid cultures; (2) tumor samples directly obtained from breast cancer patients or xenograft.

Exclusion criteria for the manuscripts were as follows: (1) use of cell lines to establish 3D organoid cultures; (2) studies considering other 3D cultures (spheroid cultures, for example); (3) no description of the organoid establishment method; (4) PDX originally established from cell lines; (5) interviews, congress and conference abstracts or posters, comments, newspaper articles and non‐English/non‐Portuguese language studies.

If there were duplicate articles using the same samples, we considered the most recent published work.

### 2.4. Risk of Bias

We evaluated the risk of bias via Science in Risk Assessment and Policy (SciRAP) [19], a web tool to assess the relevance and reliability of in vitro toxicity data, consisting of criteria to evaluate both *reporting quality* and *methodological quality* of studies. We adapted the criteria to consider organoids instead of cell lines and focused on culture components instead of drug treatment. The reporting quality comprehends 31 items. Of these, we selected 10 items that were adequate to the present analysis, consisting of (1) the test system (e.g., cell line/cells/tissue/organ/embryo/subcellular fractions) was described; (2) the source of the test system was stated; (3) the number of cell passages of the cell line used, was stated.; (4) composition of media was described, including use of serum, antibiotics, and so on; (5) Incubation temperature, humidity, and CO2 concentration were described; (6) measures taken for avoiding or screening for contamination by Mycoplasma, bacteria, fungi and virus were described.; (7) the administered dose levels or concentrations were stated; (8) cell density or number of cells used during treatment was described; (9) the duration of treatment was stated; (10) the number of replicates per dose level/concentration or the number of times the experiment was repeated was stated.

### 2.5. Statistical Analysis

The heterogeneity of the studies was assessed by I2 (%), with the null hypothesis of the heterogeneity test being that the studies are not heterogeneous, with the following interpretation: 0%–25% indicating low heterogeneity, 26%–50% moderate heterogeneity, 51%–75% substantial heterogeneity, and > 75% high heterogeneity. Forest‐plot graphs were conducted for the total number of studies and by subgroups (histological subtype and tumor type). We employed a fixed meta‐analysis model to account for variability between studies, given the anticipated heterogeneity in study populations and methodologies to calculate the overall prevalence rates along with 95% confidence intervals (CIs). The effect measure was the proportion of established organoids.

A sensitivity analysis was performed using a random‐effects meta‐analysis model to estimate the pooled prevalence rates and corresponding 95% CIs. Under this approach, individual study weights were determined according to the random‐effects model, which incorporates both within‐study and between‐study variance. The effect measure was the proportion of established organoids. In this analysis, 9 out of 10 studies were evaluated, after exclusion of data from Shu et al. 2022, who analyzed 16 samples and reported a low organoid establishment rate (32%). Analyses were performed using Stata/MP 14.0 software for Windows. Analyses were performed using Stata/MP 14.0 software for Windows.

## 3. Results

**Figure 1:**
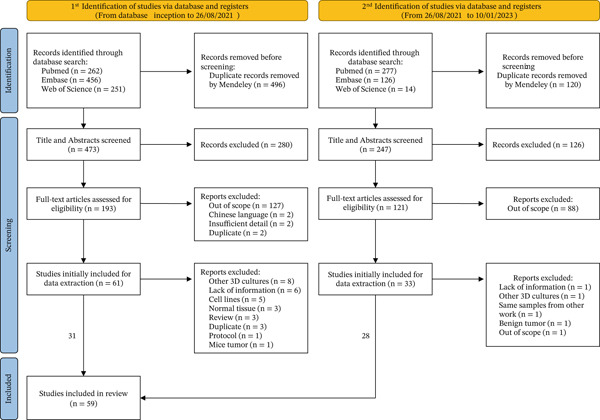
PRISMA flow diagram.

We retrieved a total of 969 titles and abstracts from PubMed, Embase, and Web of Science databases on the first search, done in date. Using Mendeley, duplicates were removed, leaving 470 nonduplicate titles and abstracts. After screening abstracts and titles, 280 studies were judged not relevant. Full texts of the remaining 190 papers were assessed, and 132 were excluded for the following reasons: 127 were out of scope, 2 were in Chinese, 2 had insufficient data, and 1 was a duplicate. Among these, 15 reviews were identified. Considering the limitations of database searches, each review was checked to identify additional studies meeting our inclusion criteria, resulting in three more publications selected for the next step (data extraction). Initially, 61 articles were selected for data extraction. Subsequently, 30 studies were excluded for the following reasons: eight involved other 3D culture types, six lacked sufficient information, five used cell lines to establish organoids, three cultured normal breast tissue, three were duplicates, three were reviews, one was a protocol, and one involved murine breast cancer. Thus, 33 studies from the first search were included in the review. To update this work, a second search was performed on [insert date], retrieving 367 titles and abstracts from the same databases. After removing duplicates with Mendeley, 247 unique records remained. Screening titles and abstracts excluded 126 irrelevant studies. Full‐text assessment of 121 papers excluded 88 that were out of scope. A total of 33 papers were initially selected for data extraction. Of these, five were excluded for the following reasons: one lacked sufficient information, one involved a different 3D culture type, one used samples from another publication, one cultured benign tumor tissue, and one was an abstract. Therefore, 28 studies from the second search were included. In total, 59 studies were included in this systematic review. The screening process is illustrated in Figure 1, and a summary of all included articles can be found in Table 1.

**Table 1:** Summary of included studies.

Reference	Collection method	Tumor source (primary or metastasis)	PDO or PDXO
Chung 2017 [20]	ND	Primary and metastasis	PDO
Sachs 2018 [12]	Surgery and biopsy	Primary and metastasis	PDO
Chatterjee 2019 [21]	Surgery	Primary	PDO
Nayak 2019 [22]	Surgery	Primary	PDO
Aganezov 2020 [23]	Surgery	Primary	PDO
Liu 2020 [24]	Surgery	Primary	PDO
Saatci 2020 [25]	Surgery	Primary	PDO/ PDXO
Silvestri 2020 [10]	ND	Primary	PDXO
Luo 2020 [26]	Surgery	Primary	PDO
Sudhakaran 2020 [27]	ND	Primary	PDXO
Padmanaban 2020 [11]	Surgery	Primary and metastasis	PDO
Nikulin 2020 [28]	Surgery	Metastasis	PDO
Senyuk 2021 [29]	Biopsy	Primary	PDO
Ge 2021 [30]	Surgery	Primary	PDO
Dhimolea 2021 [31]	ND	Primary and metastasis	PDO/ PDXO
Walsh 2014 [32]	Biopsy	Primary	PDO
Mazzucchelli 2019 [33]	Surgery and Biopsy	Primary	PDO
Goldhammer 2019 [17]	Surgery	Primary	PDO
Rosenbluth 2020 [34]	Surgery	ND	PDO
Whittle 2020 [35]	Biopsy	Primary	PDO/ PDXO
Pan 2020 [36]	Surgery	Primary	PDO
Jin 2020 [37]	Surgery	Primary	PDO
Wilde 2020 [38]	ND	ND	PDXO
Georgess 2020 [39]	ND	Primary	PDO
Campaner 2020 [40]	Surgery	Primary	PDO
Vernier 2020 [41]	ND	ND	PDXO
Urueña 2020 [42]	Surgery	Primary	PDO
Parsyan 2021 [43]	ND	Primary and metastasis	PDO/ PDXO
Signati 2021 [44]	Biopsy	Primary	PDO
Sharick 2020 [45]	Biopsy	Primary	PDO
Pauli 2017 [46]	Biopsy	Primary	PDO
Walsh 2016 [47]	Biopsy	Primary	PDO
Wang 2022 [48]	Surgery	Primary	PDO
Liu 2022 [49]	ND	Primary	PDO
Li 2021 [50]	ND	Primary	PDO
Guillen 2022 [15]	ND	Primary and metastasis	PDXO
Ye 2022 [51]	Surgery and Biopsy	Primary and metastasis	PDO
Liu 2023 [52]	ND	ND	PDO
Mo 2022 [53]	ND	ND	PDO
Liu 2022 [54]	ND	Primary	PDO
Jung 2022 [55]	ND	ND	PDO
Dwyer 2021 [56]	ND	ND	PDO
Luo 2021 [57]	Surgery	Primary	PDO
Liu 2022 [58]	Surgery and Biopsy	Primary	PDO
Yang 2022 [59]	ND	ND	PDO
Prince 2022 [60]	Surgery	Primary and metastasis	PDO/ PDXO
Carter 2022 [61]	Surgery	Primary	PDO
Li 2022 [62]	ND	Primary	PDO
Huang 2022 [63]	Surgery	Primary	PDO
Zou 2022 [64]	ND	Primary and metastasis	PDO
Donzelli 2022 [65]	Surgery	Primary	PDO
Xiao 2022 [66]	ND	ND	PDO
Mukherjee 2021 [67]	ND	ND	PDO
Chen 2021 [68]	Surgery and Biopsy	Primary and metastasis	PDO
Soria‐Bretones 2022 [69]	ND	ND	PDO
Wu 2022 [70]	ND	Metastasis	PDO/ PDXO
Shu 2022 [71]	Biopsy	Primary	PDO
Liu 2022 [72]	ND	Primary	PDO
Bhatia 2022 [73]	ND	Primary and metastasis	PDO/ PDXO

Abbreviations: ND, not described; PDO, patient‐derived organoid; PDXO, PDX‐derived organoid.

### 3.1. Concordance of HR and HER2 Expression in the Primary Tumor and the Organoid

To evaluate the concordance in hormonal receptor (HR) and HER2 expression, we checked all studies to look for data in the primary tumor and the organoid. This data was available for part of the samples evaluated in the 10 studies that appear on Table 2. Expression of estrogen receptor, progesterone receptor, and HER2 in the primary tumor sample was available in five, four, and five studies, respectively. In these studies, the percentage of positive samples varied from 27% to 100% for ER, 20% to 67% for PR, and 10% to 57% for HER2. Triple‐negative breast cancer subtypes were described in eight studies and varied from 0% to 29.4%. There were concordances between the primary sample and respective organoids for all three receptors, with kappa values of 0.692 for PR, 0.597 for ER, and 0.711 for HER2. Receptor expression reverted from positive in the tumor to negative in 19% of established organoids for ER expression, and around 31% for PR and 28% for HER2 expression. On the other hand, expression of ER, PR, and HER2 turned from negative to positive in one sample for each of the receptors. The concordance between primary tumor and organoid was at least 93% for ER negative, PR negative, and HER2 negative. Ki67 was described only in ten samples in total, with all samples being positive and remaining positive; however, there was no description of percentage of positivity (Table 2).

**Table 2:** Biomarkers concordance between tumor and organoid.

Organoid expression	Tumor expression				
	*n*(%)	*n*(%)	Total	Kappa	*p*value
	Positive (+)	Negative (−)			
ER(+)	25 (80.6)	1 (6.2)	26	0.692	< 0.001
ER(−)	6 (19.4)	15 (93.8)	21		
PR(+)	13 (68.4)	1 (6.7)	4	0.597	< 0.001
PR(−)	6 (31.6)	14 (93.3)	20		
HER2(+)	5 (71.4)	1 (4.0)	6	0.711	< 0.001
HER2(−)	2 (28.6)	24 (96.0)	26		
Ki67(+)	10 (100)	0 (0)	10	—	—
Ki67(−)	0 (0)	0 (0)	—		

### 3.2. Components of the Culture Medium

Among all the 59 manuscripts evaluated, we identified 5 different culture mediums (DMEM/F12 or Avanced DMEM/F12, DMEM, D10 medium, L‐WRN conditioned medium and Mammocult), and at least 37 different components in the culture medium, including: EGF; bFGF (basic fibroblast growth factor); FGF2; FGF7; FGF10; Noggin; neuregulin; heregulin; R‐spondin; Wnt3A; A83‐01; Y27632; SB202190; SB431542; B27 supplement; N‐acetylcysteine; nicotinamide; triiodothyronine (T3); cholera toxin; hydrocortisone; insulin; transferrin; Selenium; ethanolamine; O‐phosphoryethanolamine; heparin; isoproterenol hydrochloride; sodium pyruvate; estradiol; D‐glucose; Hepes; penicillin; streptomycin; Primocin; nystatin; gentamicin, Plasmocin and amphotericin.

The Top 10 components of the culture medium used in the 59 studies, excluding antibiotics, were, EGF (*n* = 47, 79.7%), glutamine (*n* = 39, 66.1%), Y27632 (*n* = 39, 66.1%), FGF (*n* = 38, 64.4%, considering together bFGF, FGF2, FGF7, and FGF10), R‐spondin (*n* = 38, 64.4%), N‐acetylcysteine (*n* = 38, 64.4%), Noggin (*n* = 37, 62.7%), A83‐01 (*n* = 37, 62.7%), B27 supplement (*n* = 37, 62.7%), Hepes (*n* = 37, 62.7%). These 10 components are represented in Table 3.

**Table 3:** Components of the culture medium most commonly used in the studies. EGF, epidermal growth factor; FGF, fibroblast growth factor; NAC, N‐acetylcysteine.

Reference	EstR%	EGF	Y27632	Glutamine	NAC	R‐spondin	FGF	Noggin	A83‐01	B27	Hepes
Pauli 2017 [46]^a^	66.7										
Sachs 2018 [12]	65.5	X	X	X	X	X	X	X	X	X	X
Chatterjee 2019 [21]	64.7		X								X
Campaner 2020 [40]	53.1	X	X	X	X	X	X	X	X	X	X
Padmanaban 2020 [11]	86.2	X									
Sharick 2020 [45]	54.2	X	X	X	X	X	X	X	X	X	X
Chen 2021 [68]	75		X	X	X		X				X
Guillen 2022 [15]	79.5	X	X	X	X	X		X	X		X
Huang 2022 [63]	71.4	X	X	X	X	X	X	X	X	X	
Shu 2022 [71]	31.2	X	X	X	X	X	X	X	X	X	X

^a^Pauli et al. used matrigel and mammocult, with no further factors. estR: % establishment rate.

Among the components used as scaffold for 3D culture, 55 studies used BME (basement membrane matrix) or Matrigel, four studies used 3D collagen, one study used neutralized rat tail Collagen 1, and one study used gel foam inserts. Two studies used both Matrigel and 3D collagen. One of the studies did not specify which component was used.

As a varied culture medium composition was used across the studies, we performed a qualitative comparison between studies reporting establishment rates ≥ 70% (*n* = 4) and those with lower success rates (*n* = 6). This comparison showed considerable overlap in culture medium composition between the two groups. Core components commonly used in organoid protocols, including EGF (70%), FGF family members (60%), R‐spondin (60%), Noggin (60%), A83‐01 (60%), Y27632 (80%), N‐acetylcysteine (70%), B27 supplement (50%), and Advanced DMEM/F12 (80%), were present in most studies regardless of establishment efficiency, indicating that these elements alone do not differentiate high‐ from lower establishment protocols.

To further explore potential associations between culture composition and establishment efficiency, we conducted a descriptive analysis of studies reporting establishment rates ≥ 70%. Four studies met this criterion. Within this subgroup, EGF, Y27632, and FGF family members were consistently used, and BME/Matrigel served as the scaffold in all cases. Given the small number of studies, no formal statistical analysis was performed.

### 3.3. Establishment Rate of Organoids

**Figure 2:**
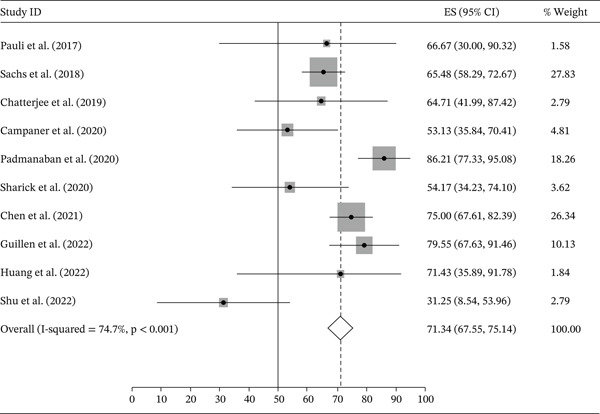
Organoid establishment rate in each study.

**Figure 3:**
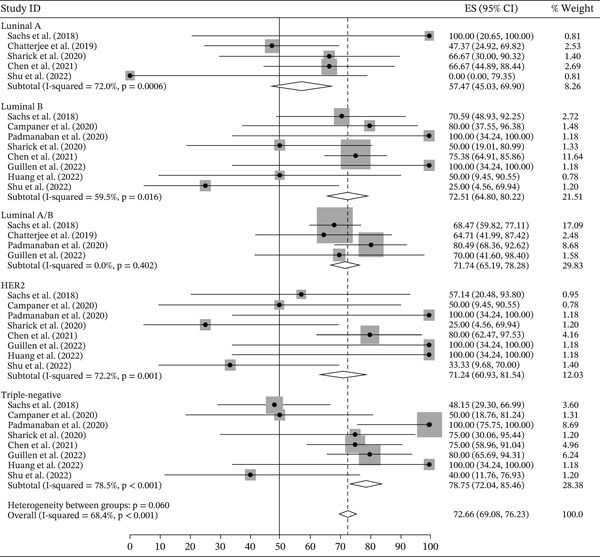
Organoid establishment rate by immunochemistry subtype.

**Figure 4:**
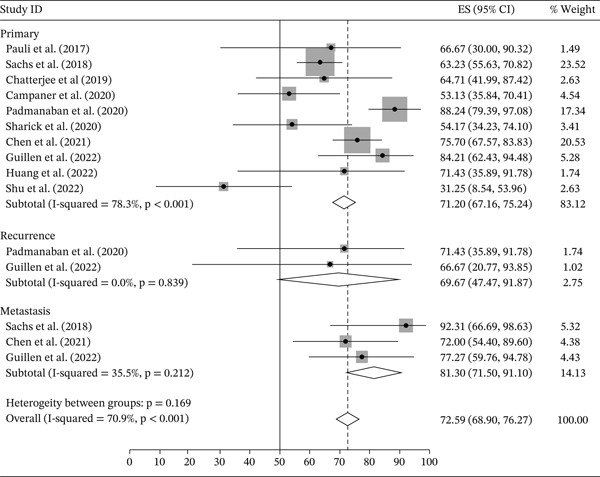
Organoid establishment rate by sample source.

Ten out of 59 studies had complete data concerning the initial number of samples and the establishment rate. The other 49 studies did not provide evaluable data and will not be further evaluated. Among the 10 considered studies, nine performed PDO and one, PDX (patient‐derived xenograft) derived organoid. To obtain cell suspensions from the whole tumor, eight studies used the enzymatic and mechanical dissociation methods and two used only the enzymatic dissociation. A summary of these 10 studies is shown in Table 4. Putting up these studies together, 504 breast cancer samples were collected, and 349 organoids established, with an overall establishment rate of 71.34% (95% CI: 67.55‐75.14), varying from 31.25% to 86.21% in the different studies. The establishment rates for each study is shown in Figure 2. The establishment rates stratified by molecular subtypes were as follows: Luminal A subtype (*n* = 45): 57.47% (95% CI: 45.03–69.90); Luminal B (*n* = 107): 72.51% (95%, 64.80–80.22); HER2 (*n* = 45): 71.24 (95% CI: 60.93–81.54); and triple‐negative (*n* = 114) 78.75 (95% CI: 72.04–85.46) (Figure 3). We next compared the establishment rate among the different sample sources (primary tumor, metastasis, or recurrence). Most studies used primary tumor samples as the source (*n* = 434) and the establishment rate was 71.20% (95% CI: 67.16–75.24). For studies that used metastasis (*n* = 60) or recurrence samples (*n* = 10) as tissue source the establishment rates were 81.30% (95% CI: 71.50–91.10) and 69.67% (95% CI: 47.47–91.87), respectively (Figure 4). However, there was a substantial heterogeneity across studies.

**Table 4:** Summary of the 10 studies that provide the initial number of tissue samples. ND, not described.

Reference	*n*samples	ER (+) *n* (%)	PR (+) *n* (%)	HER (+) *n* (%)	Dissociation method	Establishment rate (%)
Pauli 2017 [46]	6	ND	ND	ND	*E* + *M*	4/6 (66.7%)
Sachs 2018 [12]	168	117 (69.6)	103 (61.3)	21 (12.5)	*E* + *M*	110/168 (65.5%)
Chatterjee 2019 [21]	17	17	ND	ND	E	11/17 (64.7%)
Campaner 2020 [40]	32	14 (43.7)	10 (31.2)	1 (3.1)	*E* + *M*	17/32 (53.1%)
Padmanaban 2020 [11]	58	44 (75.9)	40 (68.9)	6 (10.3)	*E* + *M*	50/58 (86.2%)
Sharick 2020 [45]	24	19 (79.2)	17 (70.8)	4 (16.7)	E	13/24 (54.2%)
Chen 2021 [68]	132	ND	ND	ND	*E* + *M*	99/132 (75%)
Guillen 2022 [15]	44	12 (27.2)	9 (20.4)	5 (11.4)	E	35/44 (79.5%)
Huang 2022 [63]	7	2 (28.2)	3 (42.9)	4 (57.1)	*E* + *M*	5/7 (71.4%)
Shu 2022 [71]	16	ND	ND	ND	*E* + *M*	5/16 (31.2%)

Abbreviations: E, Enzymatic; M, mechanic.

We then repeated the meta‐analyses using a random‐effects model, after excluding one study that reported a low establishment rate of 31.25% [70] (Figures S1, S2 and S3). There was a reduction in studies′ heterogeneity for the Luminal A and Luminal B subgroups, with *I* ^2^ = 44*%* (*p* = 0.147) for Luminal A and *I* ^2^ = 30.86*%* (*p* = 0.193) for Luminal B and establishment rates of 65.9% and 75.4%, respectively.

### 3.4. Risk of Bias

**Figure 5:**
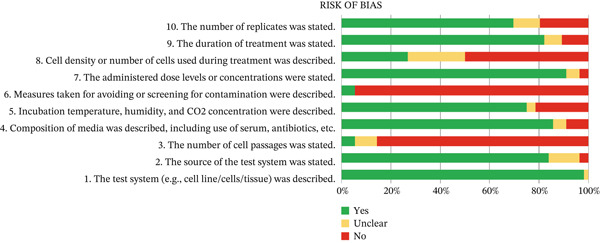
Risk of bias classification among included studies (%).

Of the 10 SciRap criteria used to assess reporting quality and potential risk of bias in articles, most items were answered in their entirety in the studies evaluated. Among the 59 studies, 52 (88.1%) provided a complete description of culture media composition, including growth factors, supplements, and administered concentrations. The origin of the biological material was also well reported, with only two studies providing partial information (Figure 5).

In contrast, several key methodological details were poorly documented. The declaration of the number of organoid passages was reported in only five studies (8.5%), whereas measures to prevent or monitor contamination by Mycoplasma, bacteria, or fungi were described in only three studies (5.1%), representing the lowest reporting rates among all evaluated criteria.

Other experimental parameters, such as administered dose levels, treatment duration, and replication strategies, were generally better reported, although inconsistencies remained across studies. Overall, these findings indicate substantial heterogeneity in methodological reporting, particularly for factors directly related to culture stability and experimental reproducibility.

## 4. Discussion

In this systematic review, we observed that the overall establishment rate of breast cancer organoids was 71%, varying across different works from 31.25% to 86.21%. The rate was similar among most BC subtypes, but tended to be lower in the Luminal A subtype. In addition, the organoid establishment rate tended to be higher when it was derived from metastasis than when it was derived from primary tumors. However, some points should be taken into consideration. There were some differences among studies in the criteria used to define successfully established organoids, and most studies do not provide clear or standardized definitions of what constitutes an “established” breast cancer organoid. An exception was Shu et al. [71], who described as positively established organoids those that could be maintained for more than three but fewer than 20 passages.

Organoid establishment rate for Luminal A tumors was 57.4%. Luminal A tumors are characterized by low aggressiveness, reduced recurrence rate, and a low proliferative index when compared with HER2‐positive and triple‐negative breast cancers. In a previous work, our group established a tumorgraft model from freshly collected breast cancer samples in nude mice to study calcitriol effects. Three breast cancer samples originated tumorgrafts, including two Luminal B with high proliferative index (Ki67 expression: 50% and 80%), and one that was HR negative and HER2 expression positive. Among three Luminal A samples, none originated tumorgrafts [74]. Although it is an in vivo model, these results corroborate the present findings of lower organoid establishment rate for Luminal A tumors.

From a biological perspective, luminal subtypes are highly dependent on hormone receptor–mediated signaling. This biological dependence may render luminal tumor cells more sensitive to in vitro culture conditions, especially when standard organoid media fail to adequately recapitulate the hormonal and paracrine cues present in the native tumor microenvironment. Among the 10 studies analyzed in this meta‐analysis, only one reported estradiol addition to the culture media, and in this case, the establishment rate for Luminal A organoids was 66.7% [68]. In this context, the intrinsically lower proliferative capacity of Luminal A tumors may limit sustained organoid growth, suggesting that subtype‐specific culture media formulations might improve establishment rate and phenotypic stability [75].

Organoids require complex culture media for their establishment and maintenance. As organoids lack mesenchymal cells, they require a scaffold, typically Matrigel, a basement membrane of laminin and collagen IV, considered the gold standard for organotypic culture. However, there are studies that solely utilize collagen 3D as a scaffold [10, 11].

Besides the membrane as support, it is necessary to enrich the medium with various growth factors or inhibitors, such as EGF, R‐spondin (Wnt agonist), Noggin (TGF‐*β* inhibitor), nicotinamide, FGF, A83‐01 (ALK inhibitor), and SB202190 (p38 mitogen‐activated protein kinase inhibitor). However, no standardized protocol was adopted across studies [12, 14, 76].

We observed that most studies used DMEM supplemented with EGF and/or FGF. Some protocols have used Neuregulin 1/heregulin (EGF family member), which may increase the growth rate of normal organoids, even though it is not essential for establishing breast tumor organoids. Nevertheless, Guillen et al. used heregulin to establish HER2 immunohistochemical subtype organoids, likely due to its association with the HER2 protein family [15–17].

The Rho kinase inhibitor, Y27632, was one of the most commonly used components in the protocols. Some authors consider the use of Y27632 in the culture medium to be essential for the establishment and growth of 3D cells [12, 15]. Y27632′s positive effects were also shown in other culture models. In 2D cultures, Y27632 combined with fibroblasts may induce cells without any genetic alteration, from different tissues, to proliferate indefinitely [77]. In addition, Y27632 may significantly reduce cell death of cultured embryonic stem cells that have a low survival rate triggered by dissociation [78].

Another factor commonly used in organoid culture was N‐acetylcysteine (NAC), a cysteine derivative, with antioxidant action. NAC appears to promote the expansion of cells ex vivo, but in organotypic culture, it is used to reduce stress on primary cells when placed in culture [9]. Guillen et al. Two thousand and twenty two used NAC specifically to culture organoids derived from donors with hormone receptor‐positive breast cancers.

We explored a potential association between culture composition and a good organoid establishment efficiency (establishment rate ≥ 70%). Within this subgroup, EGF, Y27632, and FGF family members were consistently used, and BME/Matrigel served as the scaffold in all cases. Components involved in Wnt pathway activation (R‐spondin) and TGF‐*β* pathway inhibition (A83‐01) were also frequently present. However, given the small number of studies, no formal statistical analysis could be performed. Nevertheless, this descriptive analysis highlights recurring combinations of growth factors and signaling modulators in protocols associated with higher establishment rates.

One of the concerns is the similarity between the primary tumor sample and the organoid along progressive passage numbers. However, in general, limited information is available regarding the passage number of individual organoids. There was no information for samples in nine studies included in the present meta‐analysis; however, Sachs et al. (2018) [12] reported passage number for two samples, that were cultured up to passage 19 and 21. Examining the sequencing data of these two samples, we may observe that despite the mutational signature remained similar between the primary tumor and the organoid, there was a trend to increased tumor mutational burden in the organoids. In addition, three other studies included in this meta‐analysis investigated similarity between the organoids and the primary tumors through copy number variation. In these cases, the authors report that copy number gains and losses were largely retained even after prolonged passages [12, 17, 73].

Another important aspect is whether ER and PR expression in organoids reflects the characteristics of the original primary tumor. In the present meta‐analysis, there was a substantial concordance between the primary sample and respective organoid. Despite that, ER or PR expression reverted from positive in the tumor to negative in 19% and 31%, respectively, in the established organoids. Another author, Goldhammer et al. [17], observed a progressive decrease in ER and PR expression in the organoids with higher passage numbers. These findings suggest that in vitro culture conditions may promote clonal selection, favoring the expansion of tumor subpopulations with enhanced adaptability to artificial environments.

Other persistent challenges in cell culture are Mycoplasma contamination and organoid culture cross‐contamination. Among the included studies, only three reported methods for detecting Mycoplasma, whereas the others only mentioned the use of antibiotics and antifungals, without guaranteeing the absence of contamination through specific tests. Sachs et al. [12] states that two of their samples had cross‐contamination and emphasizes the use of methods for sample quality control, such as handling them in separate locations and validating each culture through genetic sequencing. Interspecies and intraspecies cross‐contamination are quite high in cultures with cell lines, and sample authentication is essential [79–81]. However, there is no quality control protocol for organoid cultures in routine use, although Chen et al. (2020) [23] describe an NGS method capable of authenticating organoid samples and detecting cross‐contamination and Mycoplasma contamination, which is feasible for laboratory practices and cost‐effective.

Our study should be interpreted with caution, particularly when extrapolated across different breast cancer subtypes and experimental contexts. The exclusion of 49 studies due to missing quantitative data is a limitation. This fact may be partially due to the fact that to minimize selection bias, we employed a broad search strategy using high‐level terms (“breast cancer” AND “organoids”) rather than specific outcomes like “establishment rate” or “success rate”. This ensured we captured all possible data points, even from studies where the success rate was not the primary focus. The fact that 83% of the literature fails to report the raw data necessary (total primary tumors vs. successfully established organoids) is, in itself, a significant finding. It highlights a lack of standardized reporting in the field, which our study identifies and attempts to rectify by pooling the available high‐quality data. While the meta‐analysis is based on 10 studies, these represent the only existent quantitative evidence suitable for statistical pooling. Another concern is heterogeneity among the studies regarding organoid establishment rate, which was further explored in a risk of bias and a secondary analysis. This heterogeneity may be partially due to the lack of specification of passage number, cross contamination checks, and Mycoplasma infection detection. In spite of that, previous reviews have been qualitative, and a strength of our study is that it specifically quantifies the breast cancer organoid establishment rate and stratifies it by molecular subtype.

The present analysis indicates that EGF, Y27632, FGF family members, components involved in Wnt pathway activation (R‐spondin), and TGF‐*β* pathway inhibition (A83‐01), together with BME/Matrigel, may represent commonly used components for establishing breast cancer organoids. Additional aspects that should be incorporated when reporting organoid culture include specification of passage number, cross‐contamination checks, and Mycoplasma detection.

In summary, our findings demonstrate a 70% success rate in breast cancer organoid establishment, with biomarker expression (ER, PR, and HER2) largely mirroring the primary tumors. Since 20% of samples can shift toward a receptor‐negative phenotype during cultivation, continuous phenotypic characterization across passages is recommended.

## Funding

No funding was received for this manuscript.

## Conflicts of Interest

The authors declare no conflicts of interest.

## Supporting Information

Additional supporting information can be found online in the Supporting Information section.

## Supporting information


**Supporting Information 1** Material S1: PRISMA checklist.

**Supporting Information 2** Material S2: Search strategy.

**Supporting Information 3** Figure S1: Organoid establishment rate in each study with random‐effects model.

**Supporting Information 4** Figure S2: Organoid establishment rate by immunohistochemistry subtype with random‐effects model

**Supporting Information 5** Figure S3: Organoid establishment rate by sample source with random‐effects model.

## Data Availability

The data that support the findings of this study are available from the corresponding author upon reasonable request.
