# Supplementary material for: Establishment of Breast Cancer Organoids: A Systematic Review and Meta‐Analysis
Source: Int J Breast Cancer. 2026 May 10;2026:6534449. doi: 10.1155/ijbc/6534449 (PMC13158365; doi:10.1155/ijbc/6534449)
Supplement: Supplementary file 2 — Supporting Information 2 Material S2: Search strategy. [file IJBC-2026-6534449-s003.docx]

**Supp Table 1**. Search strategy

| **Database** | **Terms and combinations** |
| --- | --- |
| PubMed | ("Breast cancer"[Title/Abstract] OR "Breast cancers"[Title/Abstract] OR "Breast tumor"[Title/Abstract] OR "Breast Neoplasm"[Title/Abstract] OR "Breast tumors"[Title/Abstract] OR "Breast Neoplasms"[Title/Abstract] OR "Breast carcinoma"[Title/Abstract] OR "Breast carcinomas"[Title/Abstract] OR "Mammary cancer"[Title/Abstract] OR "Mammary cancers"[Title/Abstract] OR "Malignant Neoplasm of Breast"[Title/Abstract] OR "Breast Malignant Neoplasm"[Title/Abstract] OR "Breast Malignant Neoplasms"[Title/Abstract] OR "Malignant Tumor of Breast"[Title/Abstract] OR "Breast Malignant Tumor"[Title/Abstract] OR "Breast Malignant Tumors"[Title/Abstract] OR "Cancer of Breast"[Title/Abstract] OR "Cancer of the Breast"[Title/Abstract] OR "Human Mammary Carcinoma"[Title/Abstract] OR "Human Mammary Carcinomas"[Title/Abstract] OR "Human Mammary Neoplasm"[Title/Abstract] OR "Human Mammary Neoplasms"[Title/Abstract]) AND ("Organoid"[Title/Abstract] OR "Organoids"[Title/Abstract] OR "Organ-on-a-chip"[Title/Abstract]) |
| Web of Science | (TI=(“Breast cancer” OR “Breast cancers” OR “Breast tumor” OR “Breast Neoplasm” OR “Breast tumors” OR “Breast Neoplasms” OR “Breast carcinoma” OR “Breast carcinomas” OR “Mammary cancer” OR “Mammary cancers” OR “Malignant Neoplasm of Breast” OR “Breast Malignant Neoplasm” OR “Breast Malignant Neoplasms” OR “Malignant Tumor of Breast” OR “Breast Malignant Tumor” OR “Breast Malignant Tumors” OR “Cancer of Breast” OR “Cancer of the Breast” OR “Human Mammary Carcinoma” OR “Human Mammary Carcinomas” OR “Human Mammary Neoplasm” OR “Human Mammary Neoplasms”) OR AB=(“Breast cancer” OR “Breast cancers” OR “Breast tumor” OR “Breast Neoplasm” OR “Breast tumors” OR “Breast Neoplasms” OR “Breast carcinoma” OR “Breast carcinomas” OR “Mammary cancer” OR “Mammary cancers” OR “Malignant Neoplasm of Breast” OR “Breast Malignant Neoplasm” OR “Breast Malignant Neoplasms” OR “Malignant Tumor of Breast” OR “Breast Malignant Tumor” OR “Breast Malignant Tumors” OR “Cancer of Breast” OR “Cancer of the Breast” OR “Human Mammary Carcinoma” OR “Human Mammary Carcinomas” OR “Human Mammary Neoplasm” OR “Human Mammary Neoplasms”)) AND (TI=("Organoid" OR "Organoids" OR "Organ-on-a-chip") OR AB=("Organoid" OR "Organoids" OR "Organ-on-a-chip")) |
| Embase | ('breast cancer':ab,ti OR 'breast cancers':ab,ti OR 'breast tumor':ab,ti OR 'breast neoplasm':ab,ti OR 'breast tumors':ab,ti OR 'breast neoplasms':ab,ti OR 'breast carcinoma':ab,ti OR 'breast carcinomas':ab,ti OR 'mammary cancer':ab,ti OR 'mammary cancers':ab,ti OR 'malignant neoplasm of breast':ab,ti OR 'breast malignant neoplasm':ab,ti OR 'breast malignant neoplasms':ab,ti OR 'malignant tumor of breast':ab,ti OR 'breast malignant tumor':ab,ti OR 'breast malignant tumors':ab,ti OR 'cancer of breast':ab,ti OR 'cancer of the breast':ab,ti OR 'human mammary carcinoma':ab,ti OR 'human mammary carcinomas':ab,ti OR 'human mammary neoplasm':ab,ti OR 'human mammary neoplasms':ab,ti) AND ('organoid':ab,ti OR 'organoids':ab,ti OR 'organ-on-a-chip':ab,ti) |
