# Supplementary figures and images for: Establishment of Breast Cancer Organoids: A Systematic Review and Meta‐Analysis
Source: Int J Breast Cancer. 2026 May 10;2026:6534449. doi: 10.1155/ijbc/6534449 (PMC13158365; doi:10.1155/ijbc/6534449)

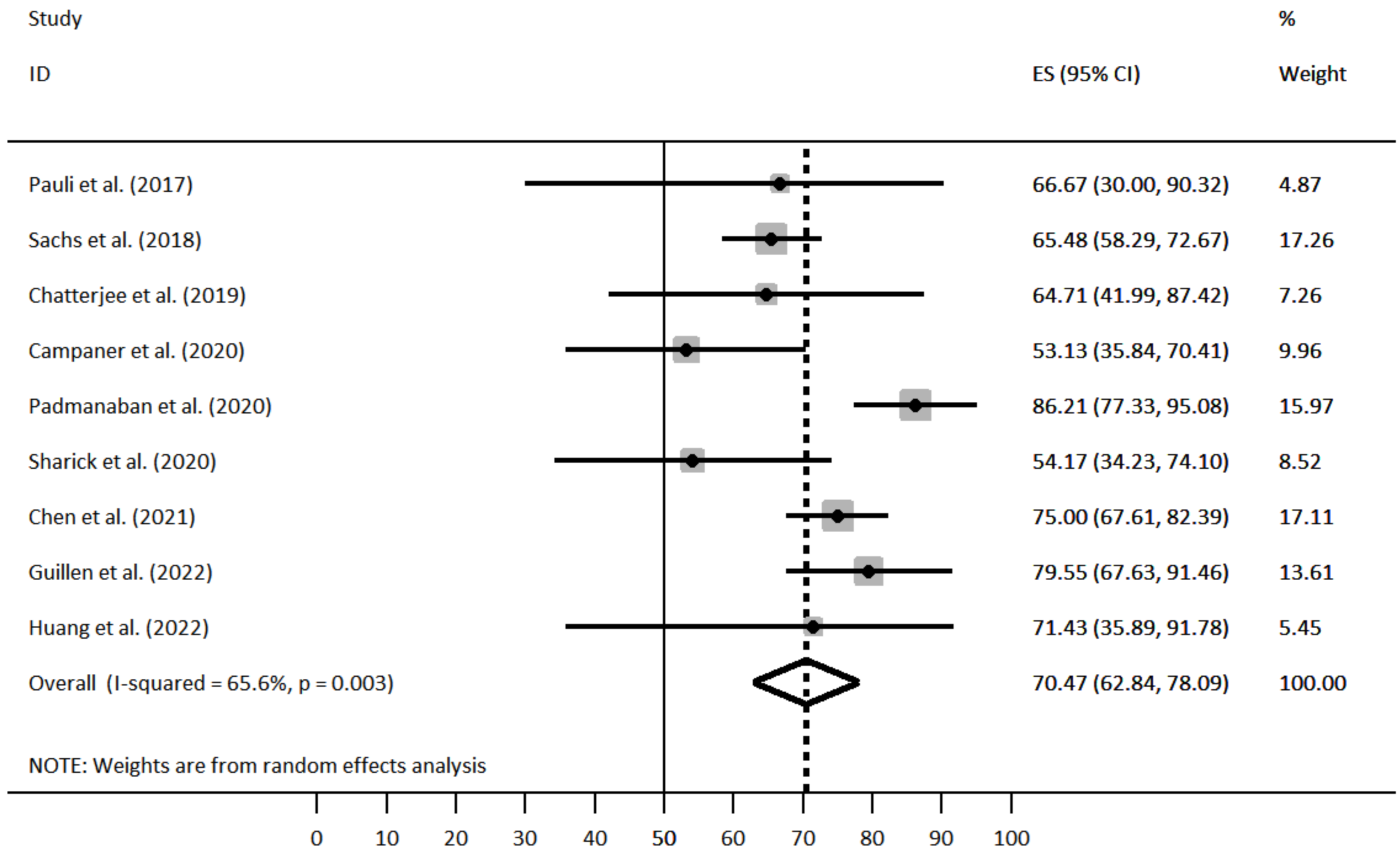

Supplement: Supplementary file 3 — Supporting Information 3 Figure S1: Organoid establishment rate in each study with random‐effects model. [file IJBC-2026-6534449-s001.pdf]

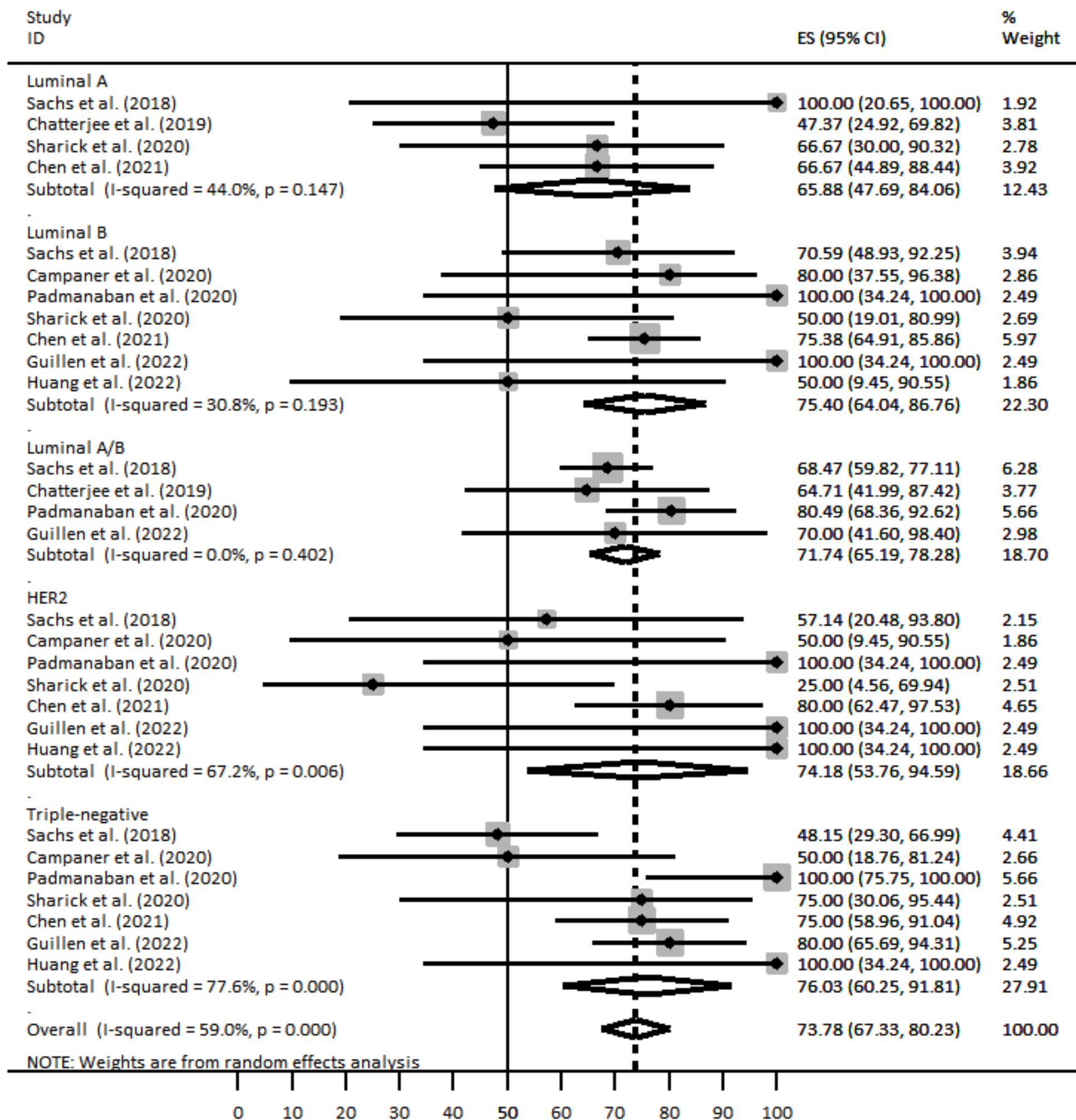

Supplement: Supplementary file 4 — Supporting Information 4 Figure S2: Organoid establishment rate by immunohistochemistry subtype with random‐effects model [file IJBC-2026-6534449-s004.pdf]

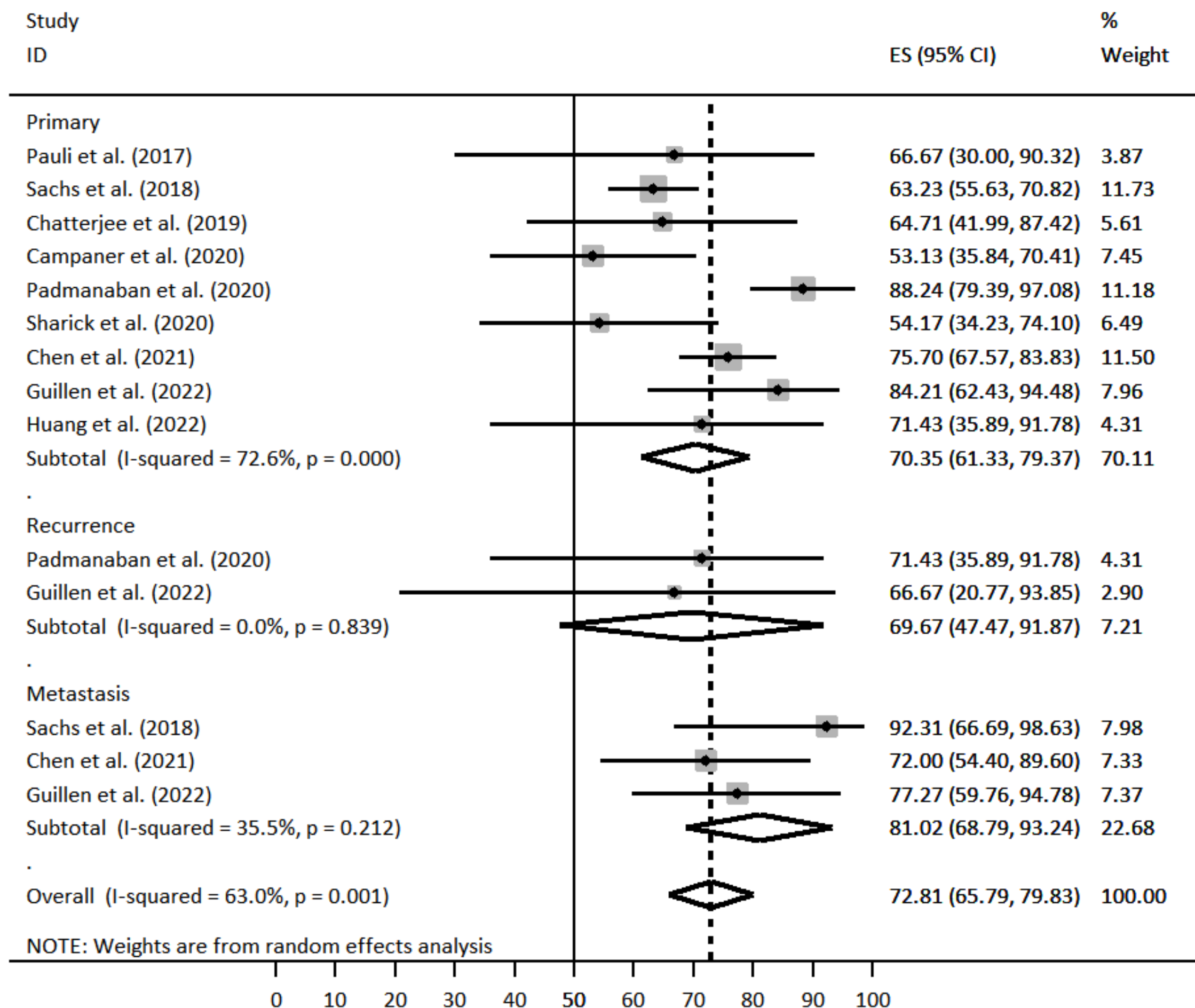

Supplement: Supplementary file 5 — Supporting Information 5 Figure S3: Organoid establishment rate by sample source with random‐effects model. [file IJBC-2026-6534449-s005.pdf]
